# Rapid and low-cost insect detection for analysing species trapped on yellow sticky traps

**DOI:** 10.1038/s41598-021-89930-w

**Published:** 2021-05-17

**Authors:** Elias Böckmann, Alexander Pfaff, Michael Schirrmann, Michael Pflanz

**Affiliations:** 1 Institute for Plant Protection in Horticulture and Forests, Julius Kühn-Institut, Messeweg 11-12, 38104 Braunschweig, Germany; 2 Leibniz Institute for Agricultural Engineering and Bioeconomy (ATB), Potsdam-Bornim e.V., Max-Eyth-Allee 100, 14469 Potsdam, Germany

**Keywords:** Data acquisition, Data processing, High-throughput screening, Image processing, Machine learning

## Abstract

While insect monitoring is a prerequisite for precise decision-making regarding integrated pest management (IPM), it is time- and cost-intensive. Low-cost, time-saving and easy-to-operate tools for automated monitoring will therefore play a key role in increased acceptance and application of IPM in practice. In this study, we tested the differentiation of two whitefly species and their natural enemies trapped on yellow sticky traps (YSTs) via image processing approaches under practical conditions. Using the bag of visual words (BoVW) algorithm, accurate differentiation between both natural enemies and the *Trialeurodes vaporariorum* and *Bemisia tabaci* species was possible, whereas the procedure for *B. tabaci* could not be used to differentiate this species from *T. vaporariorum*. The decay of species was considered using fresh and aged catches of all the species on the YSTs, and different pooling scenarios were applied to enhance model performance. The best performance was reached when fresh and aged individuals were used together and the whitefly species were pooled into one category for model training. With an independent dataset consisting of photos from the YSTs that were placed in greenhouses and consequently with a naturally occurring species mixture as the background, a differentiation rate of more than 85% was reached for natural enemies and whiteflies.

## Introduction

Worldwide, integrated pest management (IPM) is of increasing importance to limit the intake of plant protection products. The shortcoming of every IPM system is, in addition to acceptance by the grower, precise and cost-effective pest monitoring. In the case of arthropod pests, the difficulty in fulfilling is mainly the tremendous time effort to estimate populations in the crop and/or the lack of expert knowledge to distinguish relevant species^1,2^. In contrast to many other pathogens, for arthropods, not only the presence but also the population density is relevant in decision-making, which creates two major tasks for automated monitoring: precise localization and accurate determination of pest organisms in different cropping systems. For this purpose, the introduction of low-cost, time-saving, and easy-to-operate tools will play a key role in the acceptance and application of IPM in practice. Such techniques can also contribute to a broader market share of precision spraying machinery for horticultural production^3^. Most automated monitoring tools could provide maps of pest distributions, especially in phytosanitary sensitive environments such as greenhouses.

However, the development of automated detection faces the difficulty that the targets are mobile and tend to hide from view. The mobility of flying arthropods, on the other hand, has been exploited for a long time to lure pests into traps for monitoring. For some pests, studies have shown that the density of arthropods caught by a trap and the arthropod densities on crops are correlated^4–7^, making these tools a potential basis for decision-making in IPM. To date, most of these techniques on the market are very limited regarding their automated arthropod identification success rates and are mostly optimized for pheromone use in orchards (e.g., Z-Trap and TrapView). Additionally, they are costly because each trap consists of a camera and sending unit for data transfer. A low-cost solution for fast monitoring is the use of YSTs, which are well established in protected tomato cultivation and are therefore evaluated in the present study. The whitefly species *Trialeurodes vaporariorum* (TRIAVA) and *Bemisia tabaci* (BEMITA) are major pests. In central Europe, TRIAVA is the most relevant species and is usually controlled by the predator *Macrolophus pygmaeus* (MACRPY), by the larval parasitoid *Encarsia formosa* (ENCAFO), or by a combination of both. Several research studies have shown that whiteflies along with their natural predators can be monitored with YSTs^2,4,7,8^.

The best prerequisite for automatic, non-destructive, and high-throughput pest monitoring has been provided by the increasing availability of optical sensors in combination with suitable image processing tools^9–11^. With the popular image processing tool ImageJ, BEMITA and TRIAVA individuals have been counted semi-automatically by manually adjusted contrast levels, with a differentiation of insect species by their sizes^12^. The system used for taking photos, marketed as Scoutbox (Agrocares, The Netherlands), however, is expensive and heavy due to the high-end camera system plus the box construction. The authors therefore also state that more research should be performed using smartphone cameras. Still frequently used is the extraction of global features from RGB images along with supervised learning by, e.g., support vector machines (SVMs), to discriminate pests from plants or image backgrounds. This method was applied in stationary recognition systems to classify flying insects in the field^13^ and in greenhouses^14^. More recently, key point detectors were used to calculate local invariant features, which are signatures of an image representative of its structure^15^. Later, the same working group showed a high discriminability of five flying insects using local invariant features, with a BoVW classifier and a scale-invariant feature transform (SIFT) as the local image descriptor^16^. In principle, the BoVW approach is designed to achieve useful classification results even with small image datasets. Therefore, a small dataset of 100 images (ten images per class), which came from different sources, was used in training, and the method achieved good total classification rates between 47 and 90%^17^. For more differentiated species detection, region-based convolutional neural networks (CNNs) have been used to classify insects in yellow or pheromone traps^18,19^. It was shown that it is possible to distinguish between three individuals (whitefly, *Macrolophus* and *Nesidiocoris*). Systematic tests of the BoVW approach for its capability to automatically detect different insect species in common YSTs are still lacking. The influence of decay on the detection rate when individuals remain on the boards for a longer period of time, reflecting the practical situation, has also not yet been investigated.

In this study, we show an approach that can enable growers to monitor pests and natural enemies together by simply taking photos of the suspended yellow traps with a consumer camera or smartphone. Models for the detection and quantification of pest organisms from YST images were generated with machine learning algorithms trained with the help of a BoVW classifier. We consider generating high-resolution distribution maps of pest organisms a promising practical application of our approach in greenhouse production. In this context, it is particularly important that the prediction accuracy of the BoVW approach is still accurate even if some individuals have been trapped on the YST for several days. In addition, the models should provide stable results even with entirely unknown data, including other insect species as background. This situation was verified by an independent test dataset.

## Results

### Classification of the sub-images (evaluation)

The first comparative rating of insect images from the YSTs immediately after trapping (Lab0d) and after a retention time of 7 days (Lab7d) yielded partly considerable morphological differences (Fig. 4). In the BEMITA category, for example, white pairs of wings of freshly trapped animals were clearly visible, but this appearance changed towards almost complete transparency. A dark coloured centre with very blurred boundaries between the individuals and the yellow background was retained. In contrast, the TRIAVA category, which is also a whitefly species, was only slightly affected by changes over time compared to the BEMITA category. Here, the white wings were still visually distinguishable from the yellow background even after 7 days of retention. In the MACRPY category, colour changes can also be observed. Here, the body colour of MACRPY individuals changed from bright green to pale green or dark green. However, even after 7 days of exposure, the paired symmetrical antennae of the animals were still well recognizable. In the ENCAFO category, no significant differences in the subjective appearance of the insects could be detected.

The effects of varying the model parameters vocsize, colour and quantizer on the prediction accuracy and precision are shown separately for the validation of the models on unseen sub-images from the Lab0d and Lab7d datasets together (Tables 1, 2) and for the independent full scene images from the GH0-7d dataset (Tables 3, 4). Regarding the quantizer used, no considerable differences were found in terms of the class mean accuracy. Moreover, it should be noted that the use of VQ requires considerably higher computing power. Since it produced only a minor improvement in accuracy, no further comparative results regarding the quantifier are given.

**Table 1:** Recall and precision of the different models trained by k-d tree SVM-quantizer and SGD solver for different dictionary sizes (vocsize) and colour spaces (colour) based on the Lab0d und Lab7d datasets.

ID	Voc size	Colour	Recall in %										Class mean in %	
			BEMITA		ENCAFO		MACRPY		TRIAVA		BKGRND			
			0d	7d	0d	7d	0d	7d	0d	7d	0d	7d	0d	7d
163	200	Greyscale	75.97	84.79	99.83	55.56	93.47	95.31	87.07	63.14	99.13	100.00	91.09	79.76
164	200	RGB	83.33	86.64	99.66	55.56	96.73	98.44	90.99	55.63	99.13	100.00	93.97	79.25
165	200	HSV	78.68	85.71	99.83	55.56	97.14	98.44	88.91	48.12	99.13	100.00	92.74	77.57
169	500	Greyscale	72.48	82.49	99.83	55.56	95.92	95.31	90.30	61.77	99.13	100.00	91.53	79.03
170	500	RGB	81.01	82.49	99.66	55.56	97.14	96.88	92.15	53.58	99.13	100.00	93.82	77.70
171	500	HSV	79.07	82.49	99.66	54.70	96.33	98.44	86.84	42.66	99.13	100.00	92.20	75.66
			Precision in %											
163	200	Greyscale	79.03	58.97	99.83	92.86	100.00	100.00	87.27	84.47	91.94	44.23	91.61	76.11
164	200	RGB	83.33	56.63	99.83	94.20	100.00	95.45	91.63	84.02	95.80	43.40	94.12	74.74
165	200	HSV	79.92	52.99	99.83	92.86	100.00	92.65	90.38	82.46	94.21	42.59	92.87	72.71
169	500	Greyscale	83.11	60.07	99.83	92.86	100.00	100.00	87.67	83.41	91.20	33.82	92.36	74.03
170	500	RGB	84.62	55.94	100.00	94.20	99.58	83.78	92.36	85.79	93.70	33.82	94.05	70.71
171	500	HSV	77.27	50.42	99.66	96.97	99.58	90.00	91.48	82.24	92.68	32.39	92.14	70.40

TRIAVA, *T. vaporariorum*; BEMITA, *B. tabaci*; ENCAFO, *E. Formosa*; MACRPY, *M. pygmaeus*; BKGRND, Background; ID, Model ID.

**Table 2:** Recall and precision of different models trained by k-d tree SVM-quantizer and SGD solver for different dictionary sizes (vocsize) and colour spaces (colour) based on a pooled dataset from Lab0d and Lab7d (temporal pooling).

ID	Voc size	Colour	Recall in %					Class mean in %
			BEMITA	ENCAFO	MACRPY	TRIAVA	BKGRND	
163	200	Greyscale	80.00	92.51	93.85	77.41	99.16	88.59
164	200	RGB	84.84	92.37	97.09	76.72	99.16	90.04
165	200	HSV	81.89	92.51	97.41	72.45	99.16	88.69
169	500	Greyscale	77.05	92.51	95.79	78.79	99.16	88.66
170	500	RGB	81.68	92.37	97.09	76.58	99.16	89.38
171	500	HSV	80.63	92.23	96.76	69.01	99.16	87.56
			Precision in %					
163	200	Greyscale	67.86	99.09	100.00	86.33	85.71	87.80
164	200	RGB	68.31	99.24	99.01	89.26	88.72	88.91
165	200	HSV	64.30	99.09	98.37	88.11	87.19	87.41
169	500	Greyscale	69.98	99.09	100.00	86.27	81.94	87.46
170	500	RGB	68.43	99.39	95.85	90.41	83.89	87.59
171	500	HSV	61.87	99.39	97.39	88.99	82.52	86.03

**Table 3:** Reduction to four categories by pooling for training and testing (TestSet of Lab0d and Lab7d).

ID	Voc size	Colour	Recall in %								Class mean in %	
			BEM-TRI		ENCAFO		MACRPY		BKGRND			
			0d	7d	0d	7d	0d	7d	0d	7d	0d	7d
183	200	Greyscale	96.67	91.76	99.66	55.56	95.92	100.00	99.13	100.00	97.85	86.83
184	200	RGB	98.70	90.00	99.15	55.56	97.14	98.44	98.84	100.00	98.46	86.00
185	200	HSV	97.68	91.57	99.66	56.41	96.73	95.31	99.13	100.00	98.30	85.82
189	500	Greyscale	95.80	90.00	99.49	55.56	97.96	100.00	99.13	100.00	98.10	86.39
190	500	RGB	98.55	86.86	99.49	56.41	95.10	98.44	99.13	100.00	98.07	85.43
191	500	HSV	97.54	90.59	99.66	57.26	96.33	93.75	99.13	100.00	98.16	85.40
			Precision in %									
183	200	Greyscale	98.38	90.17	99.83	97.01	100.00	100.00	92.93	35.94	97.79	80.78
184	200	RGB	98.84	90.00	100.00	95.59	99.58	94.03	95.52	33.33	98.49	78.24
185	200	HSV	99.26	89.98	100.00	95.65	99.58	96.83	93.70	36.51	98.14	79.74
189	500	Greyscale	98.81	90.00	100.00	95.59	98.77	98.46	92.18	32.39	97.44	79.11
190	500	RGB	98.98	89.86	100.00	92.96	100.00	95.45	94.21	27.38	98.30	76.41
191	500	HSV	99.41	90.23	100.00	94.37	99.16	95.24	93.19	33.82	97.94	78.42

BEM-TRI is the pooled class of *B. tabaci and T. vaporariorum.*

**Table 4:** Classification results after temporal and categorical pooling. The recall and precision of the different models trained considered the categorical pooling of BEMITA and TRIAVA and the temporal pooling of Lab0d and Lab7d.

ID	Voc size	Colour	Recall in %				Class mean in %
			BEM-TRI	ENCAFO	MACRPY	BKGRND	
183	200	Greyscale	94.59	92.37	96.76	99.16	95.72
184	200	RGB	95.00	91.95	97.41	98.88	95.81
185	200	HSV	95.09	92.51	96.44	99.16	95.80
189	500	Greyscale	93.34	92.23	98.38	99.16	95.78
190	500	RGB	93.59	92.37	95.79	99.16	95.23
191	500	HSV	94.59	92.66	95.79	99.16	95.55
			Precision in %				
183	200	Greyscale	94.82	99.54	100.00	84.09	94.61
184	200	RGB	95.08	99.54	98.37	85.06	94.51
185	200	HSV	95.25	99.54	99.00	84.89	94.67
189	500	Greyscale	95.00	99.54	98.70	82.13	93.84
190	500	RGB	95.17	99.24	99.00	81.19	93.65
191	500	HSV	95.46	99.39	98.34	83.49	94.17

As previously described, the results for different dictionary sizes (vocsize) and colour spaces (colour) are compared.

### Classification without temporal and categorical pooling

The lowest average recall in the entire experiment was 77.57%. This validation value was achieved by applying the k-d tree model 165 trained with temporal but no categorical pooling and HSV converted images as well as a vocsize of 200 words on the Lab7d dataset (Table 1). The maximum recall achieved was 93.97% for model 164 applied on the Lab0d dataset and was thus approximately 3% higher than that of model 163 trained with greyscale images and the same vocsize. The maximum recall achieved by testing on the Lab7d dataset was 79.76% using greyscale images and thus slightly better than that using RGB images. In contrast, the precision values decreased after 7 days of the insects remaining on the YST by approximately 20% compared to the test results of the initialization measurement on day 0.

**Figure 1:**
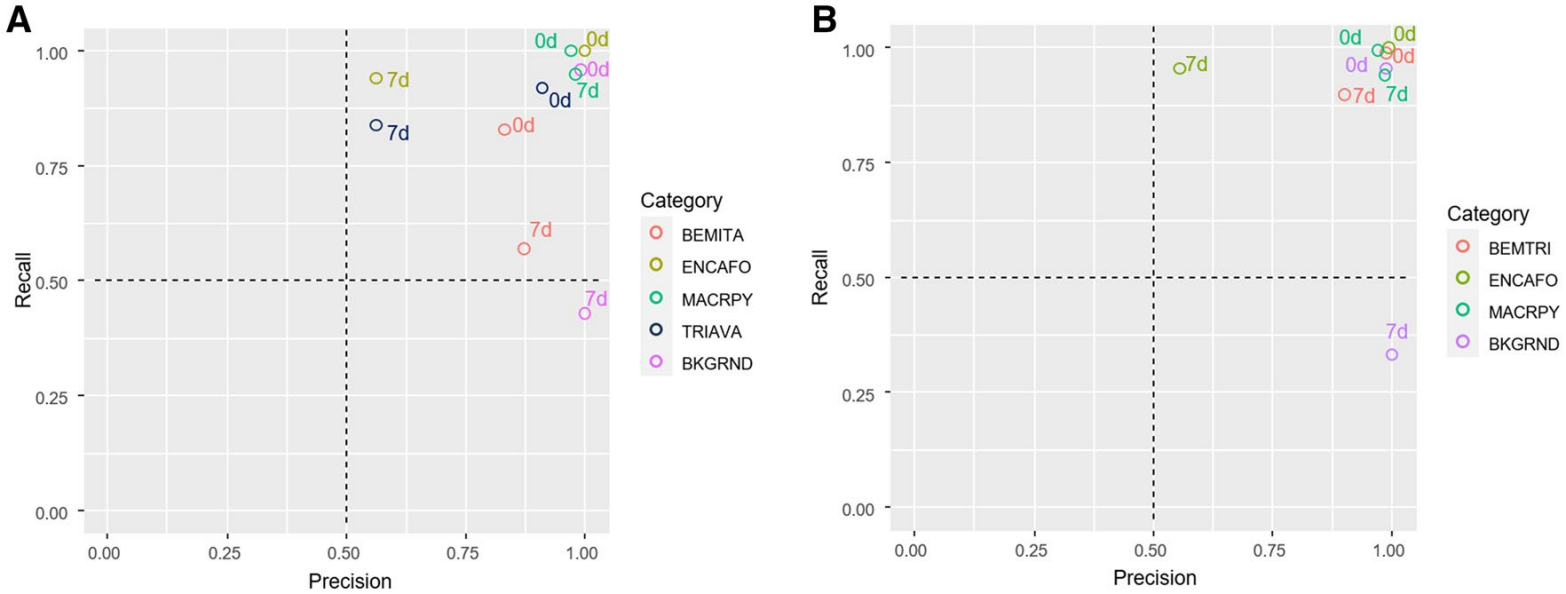
Recall-precision plots for (**A**) model 164 with no categorical pooling and (**B**) with categorical pooling. 7d = Lab7d dataset, 0d = Lab0d dataset, BEM-TRI = pooled class of *B. tabaci* and *T. vaporariorum.* The graphs were generated using ggplot in R^20,21^.

With regard to class mean accuracy, the dictionary size had no obvious influence but on the recall in individual categories. Within the individual categories, the recall of the BKGRND class was the highest, as expected. A maximum value of 99.13% was achieved without differences in colour space conversion or dictionary size. The results within the ENCAFO class were similar. Here, the best classification results were achieved with greyscale images and dictionary sizes of 200 and 500 words. For the best-performing model in terms of the overall accuracy (ID 164), the values for accuracy and precision with and without categorical pooling were plotted individually in Fig. 1A,B, respectively.

A direct comparison of the two categories BEMITA and TRIAVA (Lab0d dataset) showed that the models trained with 500 words achieved a 1% better recognition rate in the TRIAVA category, while the same value for the BEMITA category decreased by almost 2%. In terms of precision, a considerable decrease in the BEMITA and BKGRND categories was found. For the BEMITA category, the precision values were only between 50% and almost 60%; for the BKGRND class, the values were even lower, with a maximum of 44.23%. This finding indicates a less robust prediction model, especially for the categories mentioned above. However, BEMITA and TRIAVA were less well recognized than the objects in the other categories. Remarkably, the recall in the BEMITA category shows slightly increased values even after one week of the insects remaining in the trap. The highest recall was found here, with 86.64% for RGB images and a dictionary size of 200 words.

The different performances of the models with regard to feasible categorical pooling of BEMITA and TRIAVA are shown again in a recall-precision plot (Fig. 1). Essentially, this plot shows that images of the Lab0d dataset from the categories ENCAFO, MACRPY and BKGRND were detected correctly with almost 100% confidence; for BEMITA and TRIAVA, the values are above 80%. Here, it becomes apparent that the morphological changes in individual insects due to the 7 days remaining on the YST show a considerable influence on the detection rate.

The lowest detection rates were found in all other categories of images of the Lab7d dataset with the exception of MACRPY and in addition to the BKGRND class (Fig. 1A). With the exception of MACRPY, the values for ENCAFO and TRIAVA are below 60% for the recall but above 75% for the precision. For BEMITA, the recall is above 86% (100% for BKGRND), but the precisions are below 60% and 50%. After pooling the categories BEMITA and TRIAVA, the negative effects of decay could be reduced. Only the categories BKGRND and ENCAFO still show a poor detection rate with the use of the Lab7d dataset.

### Classification with temporal pooling

The results of testing after combining the Lab0d and Lab7d datasets (temporal pooling) are shown in Table 2. Compared to the test on the Lab0d dataset, the overall accuracy is approximately 4% lower on average. Nevertheless, the highest value is achieved by using RGB images and a vocsize of 200 words. With regard to the categories ENCAFO, MACRPY and BKGRND, no substantial changes are apparent. The values for the detection rate (recall) are still above 92%. With no regard to the duration of their remaining on the YST, the values from Table 2 suggest that a good detection rate can also be achieved for morphologically similar individuals such as BEMITA and TRIAVA (better than 70% for RGB images and dictionary sizes of 200 and 500 words, respectively). Considering the individual categories, it is apparent that, in particular, the precision for BEMITA was slightly lower compared to the tests on the Lab0d dataset but was improved compared to the results of the test on the Lab7d dataset (compare Table 2 with Table 1). In the BKGRND category, on the other hand, the precision could clearly be improved. Overall, the mean accuracy for temporal pooling for both the recall and precision was close to 90%, indicating high model performance.

### Classification with categorical pooling

Due to the morphological similarity of the BEMITA and TRIAVA individuals, it was reasonable to perform another test to illustrate the recognition performance of the BoVW models after combining both categories (categorical pooling). The results depending on the new category BEM-TRI (BEMITA and TRIAVA) are shown in Table 3. The maximum overall accuracy achieved was 98.46% (RGB images and a dictionary size of 200 words). Overall, the recall and precision indicated a slight gain in model performance compared to without pooling BEM-TRI. In the new category BEM-TRI, the recall values were always above 90% regardless of the insects remaining time on the YST, the used colour space and dictionary size with one exception (model ID 190 with dataset Lab7d, RGB images and a dictionary size of 500 words). The results in Table 3 also show that the values for precision were all approximately 90% or above except for the BKGRND class, which was slightly worse than without pooling BEM-TRI.

### Classification with categorical and temporal pooling

The last abstraction stage of pooling included a combination of the datasets Lab0d and Lab7d (temporal pooling), as well as the combination of the insect categories BEMITA and TRIAVA (categorical pooling). This stage is relevant under practical conditions because it cannot be determined exactly how long an arthropod has already remained on the YST at the time of sampling, and one sampling per week is realistic. After this pooling, the maximum overall accuracy was still 95.81% for RGB images and a dictionary size of 200 words (Table 4). The precision and recall for all species categories were above 90% for all the models, indicating that this pooling resulted in the most robust model.

### Test on the GH datasets—classification without categorical pooling

When non-temporally pooled models are applied to the image dataset GH0d-7d, the recall and precision results shown in Table 5 are obtained. The averaged overall accuracy for recall achieved its maximum value of 85.19% with model 164 (RGB images, vocabulary size of 200 words). The value for precision of the same model was remarkably lower at 67.11%. Here, model 163 performed best with a value of 67.11%. Particularly low values for the precision were found in the BEMITA category. Although an acceptable maximum recall of 71.64% was still achieved, the average recognition rate was only 25.44%.

**Table 5:** Classification results with no pooling for training on the Lab0d-7d dataset and testing on the GH0d-7d dataset.

ID	Voc size	Colour	Recall in %					Class mean in %
			BEMITA	ENCAFO	MACRPY	TRIAVA	BKGRND	
163	200	Greyscale	64.18	100.00	81.25	47.38	100.00	78.56
164	200	RGB	71.64	100.00	100.00	54.29	100.00	85.19
165	200	HSV	70.15	100.00	100.00	44.05	100.00	82.84
169	500	Greyscale	64.18	100.00	87.50	44.05	100.00	79.15
170	500	RGB	67.16	100.00	100.00	51.43	100.00	83.72
171	500	HSV	64.18	100.00	100.00	40.24	100.00	80.88
			Precision in %					
163	200	Greyscale	25.75	63.64	100.00	95.67	80.97	73.21
164	200	RGB	26.23	58.33	61.54	98.28	91.17	67.11
165	200	HSV	24.61	70.00	54.24	96.86	86.61	66.46
169	500	Greyscale	26.71	63.64	100.00	96.86	78.23	73.09
170	500	RGB	26.47	58.33	59.26	99.08	87.07	66.04
171	500	HSV	22.87	70.00	78.05	98.83	80.70	70.09

The reason for the low detection rate in the BEMITA category is illustrated by a confusion matrix (Table 6). From this table, it can be seen that instead of BEMITA, images are mainly classified as TRIAVA (n = 135). Category pooling is therefore quite reasonable and useful.

**Table 6:** Confusion matrix for model 164 tested on the GH0d-7d image set.

	BEMITA	ENCAFO	MACRPY	TRIAVA	BKGRND
BEMITA	48	1	0	4	14
ENCAFO	0	7	0	0	0
MACRPY	0	0	32	0	0
TRIAVA	135	4	20	228	33
BKGRND	0	0	0	0	485

### Test on the GH datasets—classification with temporal and categorical pooling

Based on the results of Tables 3 and 4, it will be shown how categorical pooling of the classes BEMITA and TRIAVA affects the recall and precision. The average overall recall increased strongly to a maximum value of 96.15% (model 184) when BEM-TRI was pooled (Table 7). The overall precision for the same model increased by 15.48% to a value of 82.59% compared to the non-pooled data. In the pooled BEM-TRI category, the maximum value for the recall was 84.60%. This value was achieved with the model trained with RGB images and using a dictionary size of 200 words (model 184).

**Table 7:** Pooling for training and testing on the GH0d-7d dataset.

ID	Voc size	Colour	Recall in %				Class mean in %
			BEM-TRI	ENCAFO	MACRPY	BKGRND	
183	200	Greyscale	79.06	100.00	90.63	100.00	92.42
184	200	RGB	84.60	100.00	100.00	100.00	96.15
185	200	HSV	80.08	100.00	100.00	100.00	95.02
189	500	Greyscale	75.36	100.00	93.75	100.00	92.28
190	500	RGB	80.90	100.00	96.88	100.00	94.44
191	500	HSV	75.15	100.00	100.00	100.00	93.79
			Precision in %				
183	200	Greyscale	99.23	70.00	100.00	83.05	88.07
184	200	RGB	100.00	63.64	78.05	88.67	82.59
185	200	HSV	100.00	77.78	82.05	84.64	86.12
189	500	Greyscale	99.46	63.64	93.75	80.97	84.45
190	500	RGB	99.75	58.33	86.11	85.39	82.39
191	500	HSV	100.00	77.78	66.67	82.48	81.73

The effects of categorical pooling are again clearly shown in the confusion matrix (Table 8). All the images in the BEM-TRI category are correctly recognized.

**Table 8:** Confusion matrix of classifications carried out with a time- and category-pooled training set (model 184) and tested on a category-pooled GH0d-7d image set.

	BEM-TRI	ENCAFO	MACRPY	BKGRND
BEM-TRI	412	4	9	62
ENCAFO	0	7	0	0
MACRPY	0	0	32	0
BKGRND	0	0	0	485

**Figure 2:**
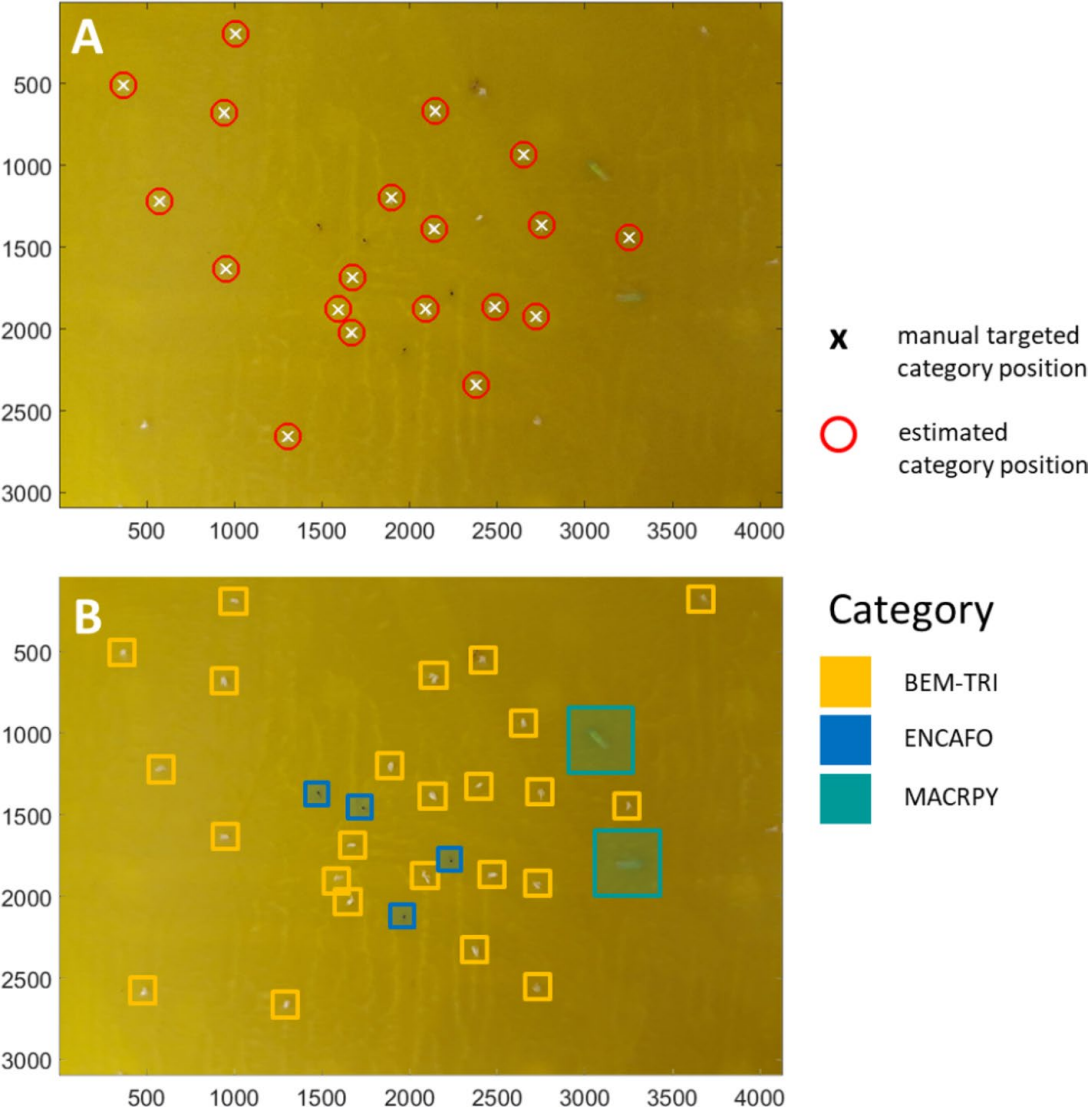
Results of spatial image classification on an image from GH0-7d dataset. (**A**) The crosses are the manually targeted positions of TRIAVA by category, and the red circles show the position of the category position estimated by the BoVW algorithm. Four additional BEMITA individuals were skipped. (**B**) Example of classification into categories BEM-TRI, ENCAFO and MACRPY. The four additional BEMITA individuals were correctly detected within the BEM-TRI category. For better clarity, the BKGRND category was excluded from the presentation.

Finally, an attempt was made to discriminate individuals applying the BoVW approach under practical conditions. For this purpose, 21 full scene images of the YST taken from the greenhouse chambers (GH0-7d dataset). Species were identified and located, and species were counted for the categories estimated above. Figure 2 shows the result of the localization. The white crosses denote the position of the manual samplings (output from ImgObjectLocator), and the red circles denote the position of the automatic detection. In Fig. 2A, manual and automatic annotation points for the category TRIAVA match nearly perfectly. Figure 2B shows a category map for the five trained categories BEM-TRI, ENCAFO and MACRPY with the corresponding colour coding.

## Discussion

Although the experiments were performed with relatively simple materials and devices, the results regarding species identification as well as the experiences with handling were encouraging. A system where growers still need to handle each yellow trap regularly, although with reduced time and knowledge requirements, does clearly not fit all growing systems. However, especially in a greenhouse environment, where a relatively large number of traps is needed to adequately reflect arthropod population developments in the crops, it has advantages compared with systems currently on the market. The cost is low because all the techniques apart from the lighting setup are already privately owned by the user, i.e., part of a standard smartphone. Therefore, the costs are much lower than those of technically highly developed stand-alone trap systems, which require components such as cameras, lighting, transmitters and power supplies. Additionally, images can be taken from all kinds of traps without dependencies on size or material. Furthermore, the additional workload of taking the pictures is limited since growers and workers in greenhouse vegetables are regularly working in the whole crop (at least once a week) and can take pictures of sticky traps on the go.

Images of the YST in this study were taken with a smartphone, or, in other words, they were not taken with a sophisticated camera system. Considering this aspect, the performance of the studied machine learning approach was surprisingly good. Clear differentiation of *E. formosa*, *M. pygmaeus* and whiteflies, including *B. tabaci* and *T. vaporariorum,* was achieved. Differentiation between the two whitefly species was not possible, mainly because the true categorization of *B. tabaci* was not possible. This finding is independent of the dictionary size, the colour space and the SVM quantizer used. Therefore, it can be assumed that the SIFT feature detector is not able to find sufficiently significant features in the images of the species, which is especially the case when identifying individuals who have been trapped on the YST for several days. A workaround could be to monitor the YST more frequently. A differentiation between BEMITA and TRIAVA may be feasible with the presented procedures if the program corrected the BEMITA counts by substitution of individuals who were already counted as TRIAVA. Because the TRIAVA procedure had very few false positives (precision > 90%, Table 4), the remaining positives of the BEMITA procedure would have very few false positives as well because misdetection can entirely be accounted for by TRIAVA. The possibility of distinguishing both species has concrete implications for pest management because the parasitoid *E. formosa* is known to be less effective on *B. tabaci* than the parasitoid *Eretmocerus mundus* and the latter vice versa to *T. vaporariorum*. Additionally, the situation for virus transmission differs for both whitefly species.

The differentiation success of the two beneficials from each other and the pest species using the GH0-7d dataset from the greenhouse environment is very promising. For instance, the recognition of *M. pygmaeus* remained very good even when high numbers of Cicadae were present on the traps, which are relatively similar in size and colour. This result indicates a certain robustness against ageing of the trapped insects, which, especially in whiteflies and *M. pygmaeus,* can result in a change in colour.

There is no doubt that deep learning approaches can achieve much better results than traditional methods, especially regarding the differentiation of very morphologically similar species. By taking images of a YST in a defined environment using a low-cost or smartphone camera and a Scoutbox, it was shown that three individuals were precisely classified on YSTs by applying region-based convolutional neural networks^18^. However, such an approach was not the aim of the present study. Here, a classical machine learning approach had to be tested for its suitability to be used with a YST because it should be considered that the size of training datasets is many times smaller than comparable datasets required to train deep neural networks.

However, it can be estimated from our results that precise differentiation between species on YSTs based on smartphone photos is possible using deep neural networks once sufficiently large datasets can be provided. The handling of the prototype is rather easy, but for professional use, the plastic box that was used to maintain a constant distance and even lighting needs to be designed with a lighting source and a clipping system that allows easy attachment to the YST. Furthermore, how robust the analyses are with regard to different smartphones and different camera types must be tested.

From the current data, experiments, and analyses, we assume that this technique is relevant for practice if 1) usability with common smartphone types is given and 2) robustness of analysis can be confirmed with images from a variety of growing situations and species mixtures on traps. Robustness against trap types from different producers would be a benefit and is already indicated due to the use of two trap types in the current study.

## Materials and methods

### Concept of the bag of visual words

The principle behind BoVW is to reduce the information content of an image in relation to a generalized set of features from many images of a specific theme (image universe). This generalized set of features is found by retrieving the most relevant information in the image universe with key point extractors and clustering the key point descriptors. The generalized features, or more specifically, the estimated cluster centres, are referred to as the visual dictionary of the image universe. With a new image from the same theme, it is then possible to approximate the nearest relationships between the features of the visual dictionary and the features extracted from that image. The frequency vector counting the number of specific relations can be seen as a footprint of the image. This frequency vector is called the bag of visual words. If we have known labels referenced to the images, we can train an image classifier just by classifying the BoVW vectors – typically with SVMs (support vector machines) or nearest neighbour approaches.

### Image classification with BoVW

**Figure 3:**
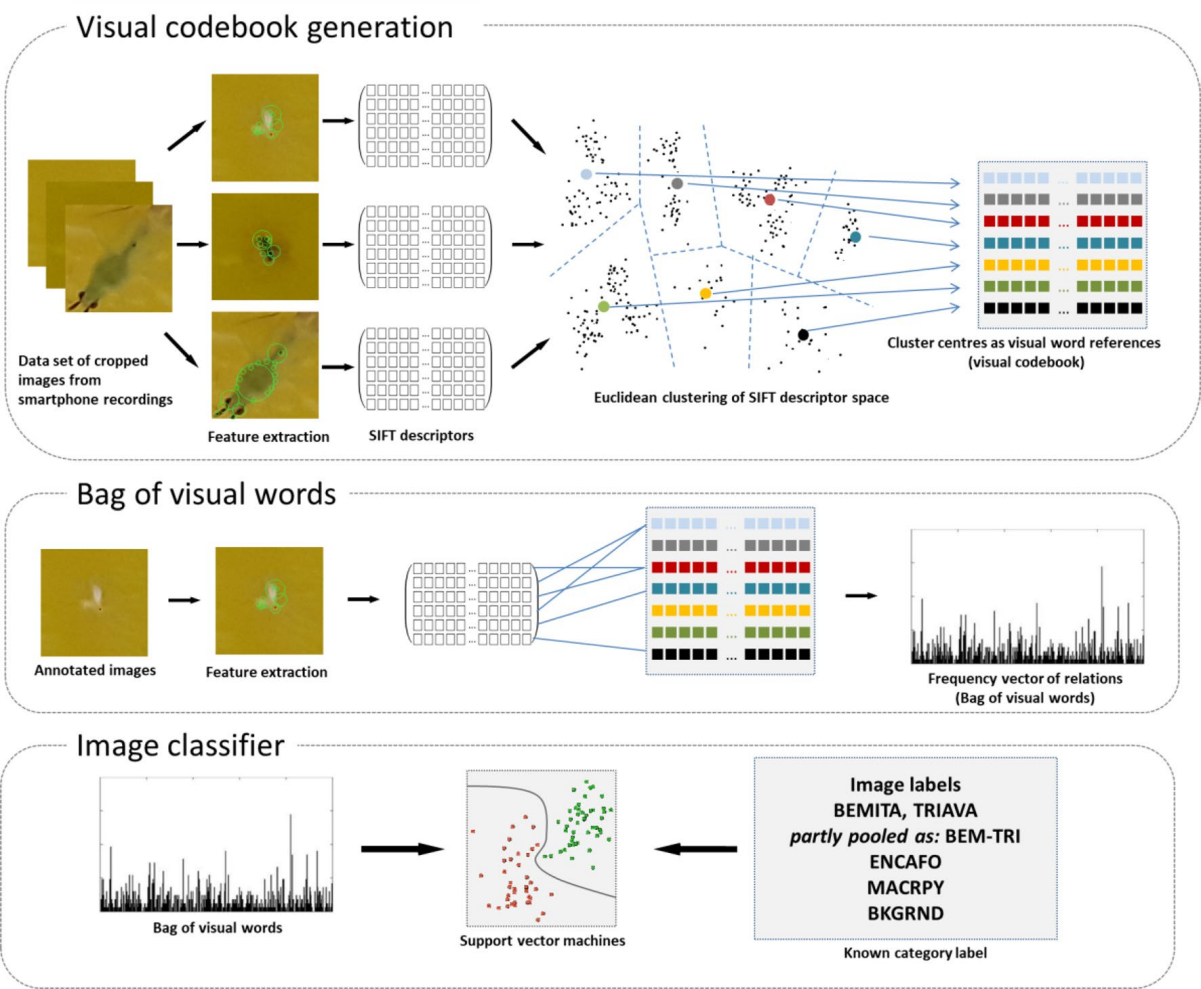
The framework of BoVW image classification. In addition to the classes BEMITA, TRIAVA and MACRPY, a pooled class BEM-TRI was generated and trained, as well as a separate yellow background class without insects (BKGRND).

In our case, the image universe is images of YSTs, and the labels are given by the arthropod species and the background of the YSTs. The image classifier was created following the study by^22^, as shown in Fig. 3. According to this concept, the first step was to extract local image features from a training dataset of numerous insect images with a SIFT key point descriptor. SIFT is a blob detector invariant to scale and rotation and a standard algorithm for key point detection and description^23^. Based on unsupervised k-means clustering, Euclidean cluster centres within the feature spaces were then calculated. The cluster centres form the visual dictionary, which means that the code words constituting the dictionary represent the average Euclidean centre of the key point descriptors that belong to one of n clusters calculated by the average Euclidean dissimilarity from cluster to cluster. This mapping of new input data into one of the clusters by looking for the nearest neighbour was tested by vector quantization (VQ) and space partitioning in a k-dimensional tree structure (k-d tree). In contrast to the linear approximating process of VQ, k-d tree uses a hierarchal data structure to find the nearest neighbours by recursively partitioning the source data along the dimension of maximum variance^24^. To build the visual dictionary, we used our own classification framework based on SIFT key point detectors, which was provided by the VLFeat toolbox (version 0.9.21) for MATLAB^24^. The dictionary size (vocsize), e.g., the number of code words (or cluster centres), was systematically varied between 200 and 500.

In the second step, local image characteristics were again extracted from the insect images—this time with known labels—with the same key point extractor and compared with the references from the visual dictionary. Key point descriptors of new images were related with an approximate nearest neighbour search to construct frequency vectors. The frequency vectors were stored as bags of visual words. Combined with known labels on the images, these frequency vectors were finally used to calibrate the SVM image classifier.

Finally, support vector machines were used to calibrate the image classifier with a linear kernel using BoVW vectors with known labels as input. SVM modelling was performed with a stochastic gradient descent (SGD) solver, which minimizes the primal optimization problem of the SVM, particularly for very small objects.

### Preparation of the yellow sticky traps

*T. vaporariorum* (TRIAVA) and B. tabaci (BEMITA) were available in separate rearings on Baylee F1 tomato plants grown in soil greenhouse chambers. E. formosa (ENCAVO) and M. pygmaeus (MACRPY) were purchased from Katz Biotech (Baruth, Germany). To trap the different insects, YSTs (IVOG, biotechnological systems GmbH) 10 × 25 cm in size were separately used for each species. YSTs were put into the rearing cages for two minutes to trap a certain number of whiteflies. For trapping, the M. pygmaeus and E. formosa YSTs were put into a plastic box together with the respective carriers of E. formosa and M. pygmaeus as delivered by Katz Biotech. A YST with both TRIAVA and BEMITA trapped on it was produced to test whether discrimination of these very similar species within the Aleyrodidae family is possible.

**Figure 4:**
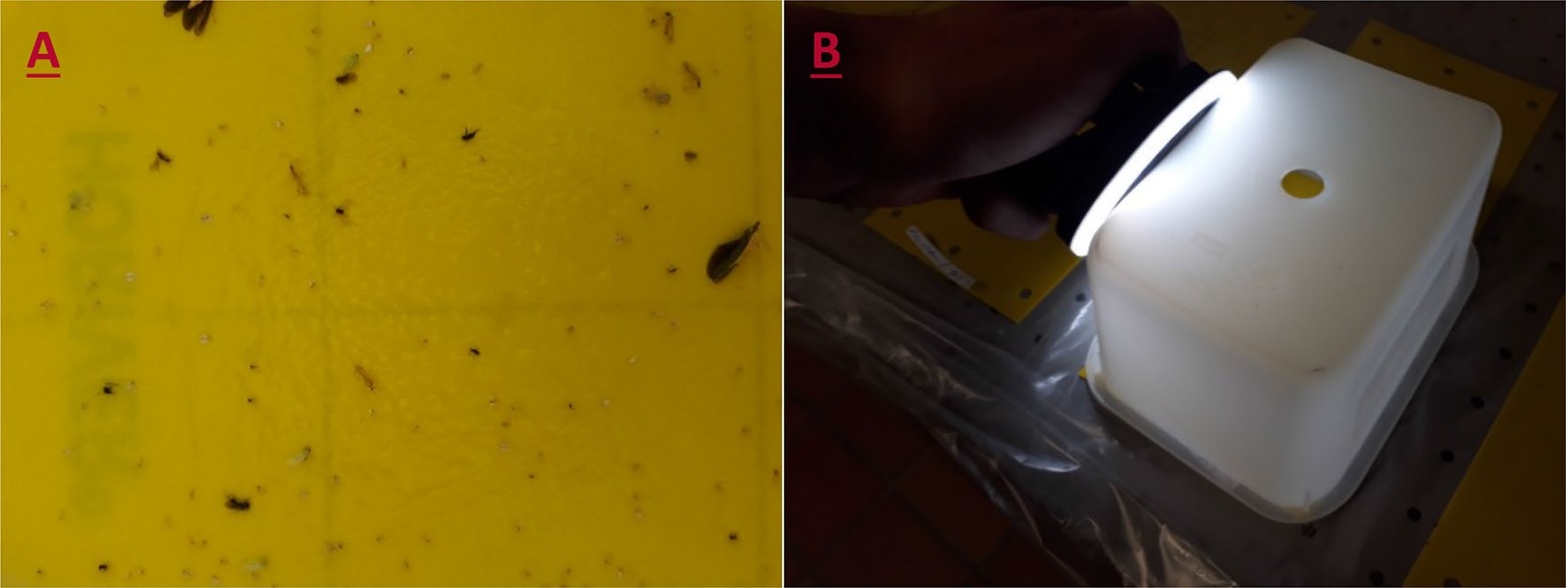
(**A**) Example of a yellow sticky trap that was taken weekly in the greenhouse chambers. This figure illustrates the realistic diversity of species present in the traps, including aphids, cicadellids, and Diptera. (**B**) To reduce light reflections on the YST surface, a plastic box and a ring light were used to capture the images.

In addition to the abovementioned YSTs in the greenhouse, Horiver YSTs (Koppert Biological Systems) were used in the same manner. The YSTs were positioned at the height of the tip of the plants between double rows in a vertical alignment. In each of four soil greenhouse chambers (40 m^2^), one YST was installed, and in a larger greenhouse (170 m^2^), two YSTs were installed. These YSTs were removed weekly, images were taken, and fresh YSTs were installed. This resulted in a trapped species mix of TRIAVA, BEMITA, MACRPY and ENCAFO (GH0-7d), of which TRIAVA occurred naturally and the beneficials MACRPY and ENCAFO were introduced. Only BEMITA did not occur naturally and was introduced into the greenhouse. To produce BEMITA catches for the GH0-7d dataset, four YSTs were put into a BEMITA rearing cage for 2 min just before they were installed in greenhouse chambers for a duration of one week. This means that all BEMITA catches within the species mix of the GH0-7d dataset were exactly 7 days old when the images were taken. In these traps, a realistic number of other insects, including mainly other hemipterans, such as aphids and cicadellids, and Diptera, were present (Fig. 4A). These other species belong to the background and were not further differentiated.

### Standardized image acquisition

To develop a functioning object recognition algorithm, images of YSTs need to be taken not only in a standardized procedure but also under conditions comparable to practical tomato cultivation. The images were taken with a “Galaxy A3” smartphone (Samsung 2017). To achieve uniform illumination without reflections on the YST surface, the dim plastic freezer box “domino” (Rotho, Würenlingen) was used as shielding (Fig. 4B). The chosen plastic box had internal dimensions of 13.8 cm × 9.8 cm and a height of 7.3 cm. In the middle of the bottom part, a hole 1.3 cm in diameter was drilled. The lid was removed, and the box was positioned upside down on the YST. The smartphone was positioned on the upside-down box in such a manner that the image could be taken through the previously drilled hole. In this way, the distance from the YST surface to the smartphone lens was determined by the height of the box (7.3 cm). As a light source, an LED selfie ring light (Mettle, Changzhou) was used and positioned on the long side of the box. The focus of the smartphone was adjusted to the YST surface, and from a series of images, the image with the sharpest contours was selected.

**Figure 5:**
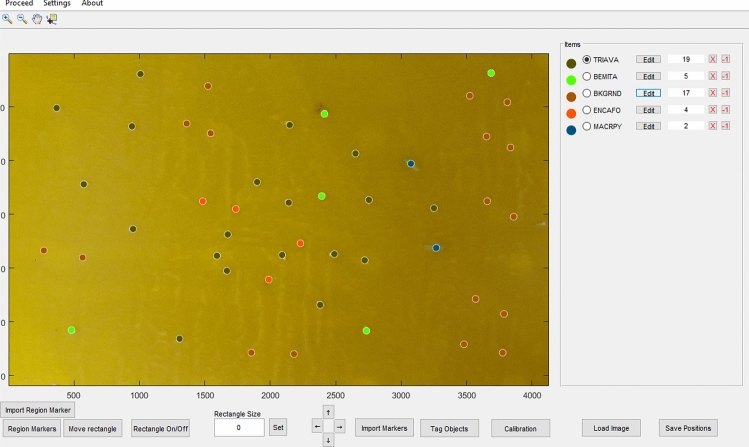
Manual labelling of the YST by the “ImgObjectLocator” software. With this tool, individuals can be marked and located in a YST. Manual labelling then provides automatic segmentation of the original image to sub-images of species, which finally gives the training data.

Training and testing of the image classifier. Initially, all the trapped individuals visible on the smartphone images of the YST were manually marked and labelled with the use of a custom MATLAB script (ImgObjectLocator Ver 0.35, MATLAB 2016b, MathWorks). The marking was done by digitizing a point in approximately the middle of each individual, from which an exact position in the image was determined and saved (Fig. 5). The image datasets for training and testing the BoVW classification models were then built from the original images by cropping sub-images with a size of 201 × 201 pixels according to the marker positions and labels.

In total, n = 5,866 images of insects were generated in this way from n = 166 YST images immediately recorded after trapping (Lab0d). Another n = 1,435 images of single insects were generated after 7 days (Lab7d) to account for decaying processes of the insects trapped on n = 87 YSTs. A completely independent dataset was generated from images of another n = 21 YSTs. From n = 1011 markers set in the ImgObjectLocator application, images were cropped to finally validate the BoVW models.

**Figure 6:**
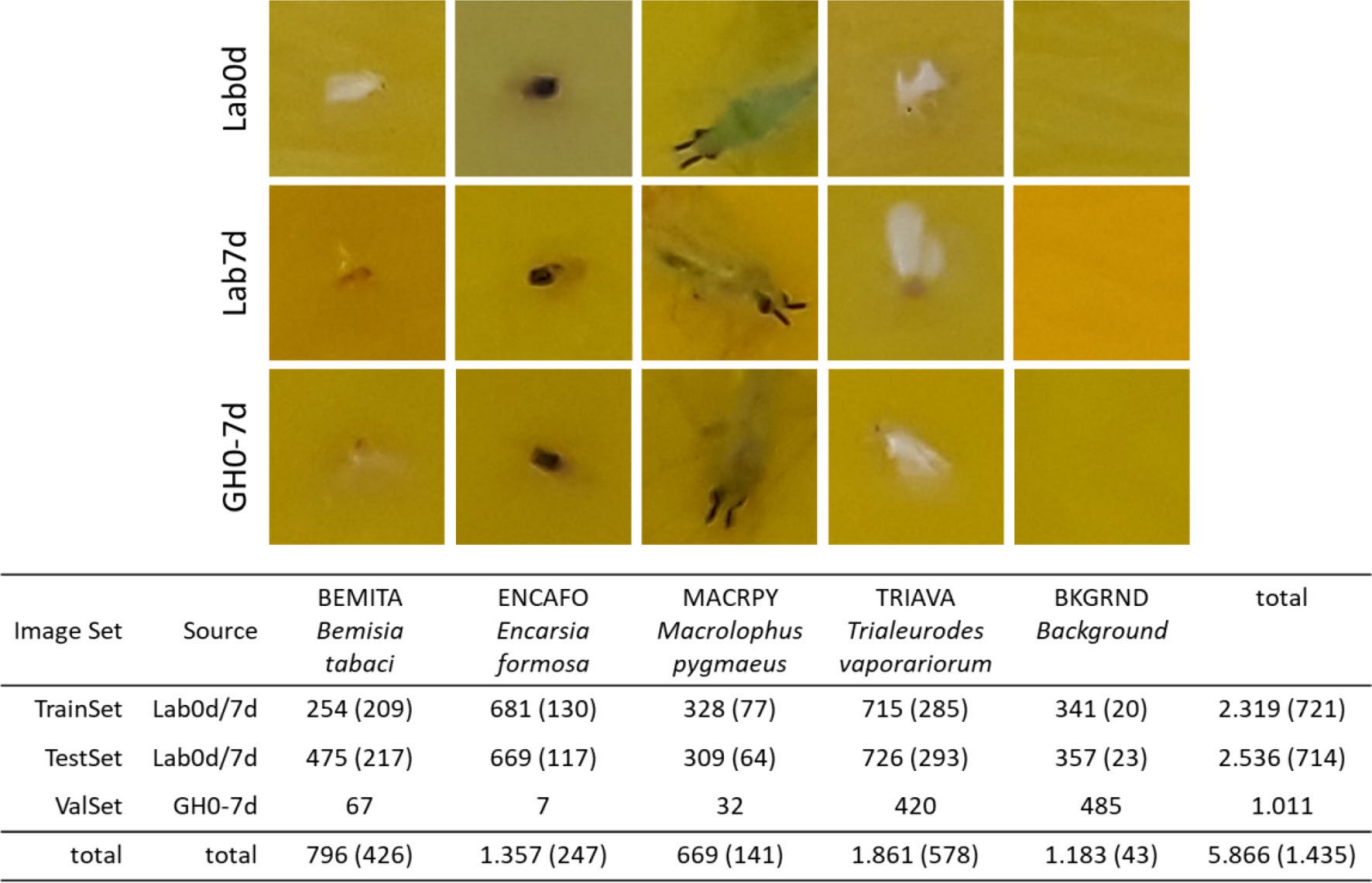
Overview of the sub-images and trained categories for the Lab0d and Lab7d datasets. The table shows the number of images provided in the corresponding datasets for training (TrainSet), testing (TestSet) and validation (ValSet). For the Lab7d dataset, the values are shown in brackets.

In summary, a dataset of four insect categories was generated for training and testing the BoVW models (Fig. 6). Such integration of categories containing only the target objects usually yields high detection rates under the assumption that it is sufficient to present a limited universe of subsequent prediction classes. This assumption is often true under defined laboratory conditions. In practice, such as insect detection under natural conditions, the detection performance would decrease and a higher number of false positives would be generated. To reduce this effect, we introduced an additional class, BKGRND, as the background or “garbage” class, which aims at the background of the yellow sticky trap (YST). The background training images were taken in the same way as the insect training images, but at locations where no insects were present. In the case of a low prediction probability for an insect class, a match with BKGRND would be high. If no background class were defined, the background of the YST would be shared among the other distinct classes, and sliding window prediction of the YST would not be possible. There was no need for an additional “garbage” class that would include other insect species because all insect species present in the greenhouse were within the prediction class space.

### Hyperparameter optimization, training and testing

To estimate the influence on the prediction accuracy, different input parameters were systematically varied. These include the dictionary size for codebook generation (vocsize), the colour space of the input images (greyscale, HSV and RGB) and the quantization on the SVMs (quantizer). An optimization and limitation of these input parameters was previously carried out by pre-testing using the Lab0d dataset. To test the impact of different colour spaces, converted 8-bit greyscale and HSV (hue, saturation and value) images were used to train the BoVW models in addition to the original 8-bit RGB images. The colour space transformations were conducted with the MATLAB built-in function﻿s rgb2gray and rgbhsv^25^. The systematic pre-test of parameter optimization finally resulted in 12 models for validation on a dataset of sub-images and for final testing. All of these models were based on the k-d tree quantizer, including three models each for colour space and dictionary size. Models with IDs from 163 to 165 and 169 to 171 were trained and tested with the full dataset of Lab0d and Lab7d (temporal pooling during model training) plus separate categories for BEMITA and TRIAVA (no categorical pooling during model training). During the training phase of the models with IDs 183–185 and 189–191, categorical pooling for the classes BEMITA and TRIAVA was carried out in addition to temporal pooling. Another six models were tested based on VQ (model IDs 160–162 and 166–168 with temporal pooling and 180–182 and 186–188 with temporal and categorical pooling). Each step was performed with a random split of the training data of 75% for modelling and 25% for optimization. Only the final output models were then evaluated on the independent test set.

### Model evaluation

To evaluate the performance of the image classifier, we calculated the parameter precision, recall and overall accuracy from the independent test set.

$${\text{Precision}} = \frac{{{\text{TP}}}}{{{\text{TP}} + {\text{FP}}}}$$

$${\text{Recall}} = { }\frac{{{\text{TP}}}}{{{\text{TP}} + {\text{FN}}}}$$

Here, TP is the number true positives, which refers to the number of items correctly labelled as belonging to the respective class, and FP is the number of false positives that are incorrectly labelled belonging to the respective class. In contrast, TN is the number of true negatives, which refers to the number of items correctly labelled as not belonging to the respective class, and FN is the number of false negatives, which refer to items incorrectly labelled as not belonging to the respective class. The precision is the fraction of relevant categories that were retrieved over the total number of relevant categories. The recall is the fraction of relevant categories that were retrieved over the total number of relevant categories. The class mean accuracy refers to the proportion of the predictions that the model estimated correctly, which was calculated as class means.

### Ethical approval

This article does not contain any studies with human participants or animals (vertebrates) performed by any of the authors.

## Data Availability

The code used to identify the examined insect species is not freely available.
