# Publisher Correction: Rapid and low-cost insect detection for analysing species trapped on yellow sticky traps

**DOI:** 10.1038/s41598-021-92251-7

**Published:** 2021-06-14

**Authors:** Elias Böckmann, Alexander Pfaff, Michael Schirrmann, Michael Pflanz

**Affiliations:** 1 Institute for Plant Protection in Horticulture and Forests, Julius Kühn-Institut, Messeweg 11‑12, 38104 Braunschweig, Germany; 2 Leibniz Institute for Agricultural Engineering and Bioeconomy (ATB), Potsdam‑Bornim e.V., Max‑Eyth‑Allee 100, 14469 Potsdam, Germany

Correction to: *Scientific Reports* 10.1038/s41598-021-89930-w, published online 17 May 2021

The original version of this Article contained an error in Figure 1, where panels (A) and (B) were duplicated. The original Figure 1 and accompanying legend appear below.

**Figure 1:**
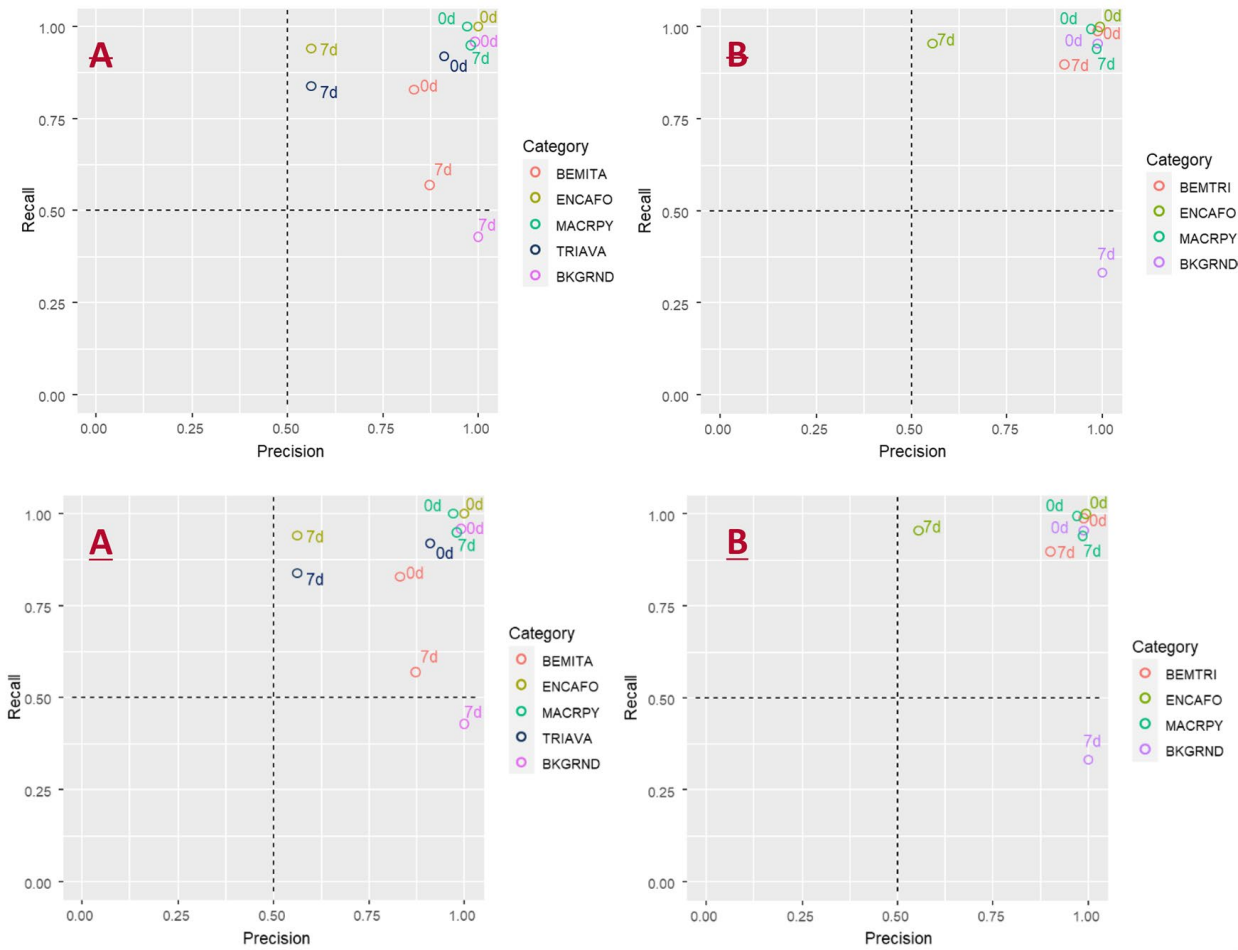
Recall-precision plots for (**A**) model 164 with no categorical pooling and (**B**) with categorical pooling. 7d = Lab7d dataset, 0d = Lab0d dataset, BEM-TRI = pooled class of *B. tabaci* and *T. vaporariorum.* The graphs were generated using ggplot in R^20,21^.

The original Article has been corrected.

